# Reconstructing the Geomagnetic Field in West Africa: First Absolute Intensity Results from Burkina Faso

**DOI:** 10.1038/srep45225

**Published:** 2017-03-28

**Authors:** Lisa Kapper, Fabio Donadini, Vincent Serneels, Evdokia Tema, Avto Goguitchaichvili, Juan Julio Morales

**Affiliations:** 1 National Autonomous University of Mexico (UNAM), Institute of Geophysics, National Archeomagnetic Service, Campus Morelia, Mexico, Morelia, 58190, Mexico; 2 University of Fribourg, Fribourg, 1700, Switzerland; 3 Università degli Studi di Torino, Dipartimento di Scienze della Terra, Turin, 10125, Italy

## Abstract

We present absolute geomagnetic intensities from iron smelting furnaces discovered at the metallurgical site of Korsimoro, Burkina Faso. Up to now, archaeologists recognized four different types of furnaces based on different construction methods, which were related to four subsequent time periods. Additionally, radiocarbon ages obtained from charcoal confine the studied furnaces to ages ranging from 700–1700 AD, in good agreement with the archaeologically determined time periods for each type of furnace. Archaeointensity results reveal three main groups of Arai diagrams. The first two groups contain specimens with either linear Arai diagrams, or slightly curved diagrams or two phases of magnetization. The third group encompasses specimens with strong zigzag or curvature in their Arai diagrams. Specimens of the first two groups were accepted after applying selection criteria to guarantee the high quality of the results. Our data compared to palaeosecular variation curves show a similar decreasing trend between 900–1500 AD. However, they reveal larger amplitudes at around 800 AD and 1650 AD than the reference curves and geomagnetic field models. Furthermore, they agree well with archaeomagnetic data from Mali and Senegal around 800 AD and with volcanic data around 1700 AD.

Archaeomagnetism, the application of palaeomagnetism to the study of archaeological artifacts, has been widely used to reconstruct the Holocene geomagnetic field. A large number of archaeomagnetic data obtained from several countries worldwide has been used so far for the calculation of palaeosecular variation (PSV) curves, e.g., the Balkan curve^1^, the Spanish intensity curve^2^, the European PSV curve^3^, and for the construction of geomagnetic field models (GFMs), e.g., SHA.DIF.14k^4^, Archeo FM^5^, CALS10K.1b^6^. One of the most common applications of archaeomagnetism is archaeomagnetic dating, in which the intensity and direction of the ancient geomagnetic field, recovered from an artefact, are compared to the reference PSV curves and GMFs, in order to estimate the artefact’s age.

Most available archaeomagnetic data come from the Northern Hemisphere and especially from Europe^7^. The acquisition of new data from regions that, up to now, are still poorly covered, will greatly improve the quality of PSV curves and GFMs, will give insight into local features of the geomagnetic field, will improve the geographic distribution of the global compilation of dipolar moments, and will remove eventual biases caused by the uneven geographical distribution of the reference data. One of these poorly covered regions is Africa, from where only 46 intensity data for the last 2000 years are reported in the most updated Geomagia50.v3 database, which is one of the largest archaeomagnetic databases^7^ (July 2016). For West and North Africa, the only available intensity data come from Mali, Morocco, Senegal and Tunisia^8^,^9^,^10^,^11^. Mitra *et al*.^11^ investigated well-dated pottery samples from Senegal and Mali from the last two thousand years, and obtained reliable archaeointensity data from this period. Gomez-Paccard *et al*.^10^ investigated six furnaces from Morocco and Tunisia, which were dated to the 9th and 15th century AD. Casas *et al*.^9^ measured enameled tiles from Moroccan tombs precisely dated to 1588–1603. Kovacheva^8^ also investigated samples from Morocco covering the first half millennium.

Special geomagnetic features, such as archaeomagnetic jerks^12^, or archaeomagnetic intensity spikes^2^,^13^,^14^, were observed in Europe, the Middle East and on the Canary Islands. Up to now, data from Africa is still too sparse to infer if such field features randomly appeared at different locations or moved around. Nevertheless, some studies observe similarities of field behavior between Africa and Europe^10^,^11^,^15^. Field features observed by or inferred from satellite data, such as the South Atlantic Anomaly (SAA), an area of low magnetic field strength, which spans the southern Atlantic Ocean, or equatorial flux spots, which are regions at the surface of the liquid core with unusually large intensity, are hardly brought into context with archaeomagnetic data. Tarduno *et al*.^16^ investigated the decay of the dipole geomagnetic field intensity over the last 160 years and its relation with the SAA. They used an archaeomagnetic record from South Africa in order to better understand the field morphology under the African continent. Therefore, archaeomagnetic data from areas close to the equator are of special interest to better understand these characteristics of the geomagnetic field.

We present here the first archaeointensity results from 17 iron furnaces from Burkina Faso, excavated at the archaeological site of Korsimoro. The archaeological context and the archaeodirectional records of the same structures have previously been studied and published by Donadini *et al*.^15^. The investigated furnaces cover a production period of 1000 years, from 700 to 1700 AD, and are a unique source of data of geomagnetic field variations in this period. The new archaeointensity data together with the previously published directional results aim to offer an important insight on the secular variation in West Africa during the last millennium.

## Rock magnetic and demagnetization results

In this section we briefly report the rock magnetic and demagnetization results previously obtained by Donadini *et al*.^15^. Furnaces from technique T1 and T4 exhibit brown to black colors. Rock magnetic measurements indicate that these furnaces have magnetite as main magnetic carrier. Furnaces from technique T2 and T3 are red to brown colored. The red specimens especially show mixtures of high and low coercivity minerals in rock magnetic experiments. These findings indicate a mixture of magnetite and hematite present in specimens from these furnaces. Additionally, a high coercivity stable low temperature (HCSLT) component with a Curie temperature of 200 °C was observed in some specimens. Two thirds of the thermomagnetic curves are either reversible or nearly reversible within 20% difference of their initial and final room temperature susceptibility values.

In total, 806 specimens from 29 furnaces were demagnetized either thermally (TH) or with an alternating field (AF). In general, they exhibit a single magnetic component with a weak viscous overprint that is removed by fields of 5 mT or temperatures of 100 °C. Sample and furnace averages with *α*_95_ ≥ 6.5° were rejected, which gave rise to the rejection of ten out of the 29 furnaces. In general, declinations range from N to NW or N to NE, and inclinations from shallow normal to shallow reversed, and in the case of T4 they are steeper, between 20–30°. These values are in agreement with the expected directions for the Holocene in Burkina Faso compared with GMFs and PSV curves. Results from T1 show clustered sample directions, but furnace averages with large dispersion for all furnaces. This dispersion is probably caused by displacement of the furnace walls after the acquisition of the ChRM. A reconstruction of the original directions was unsuccessful, in most cases, because the furnace parts have been moved randomly.

## Archaeointensity results

### Arai diagrams and their statistics

In general, a temperature interval from 100 °C to 470 °C, encompassing 12 temperature steps, was used for the principle component analysis (PCA)^17^. To guarantee the high quality of the obtained results, we applied two classes of selection criteria: soft and strict (Tables 1 and 2, adapted from Shaar *et al*.^18^). After the application of the soft selection criteria we accepted 77 of 120 specimens, and averaged them to 14 furnace values (Table 3). Two of the 77 specimens were accepted, although they did not pass the FRAC criteria; nevertheless, their values agree very well with sister specimens. With the strict criteria we accepted 35 specimens in total and obtained intensities of four furnaces (Table 4).

**Figure 1:**
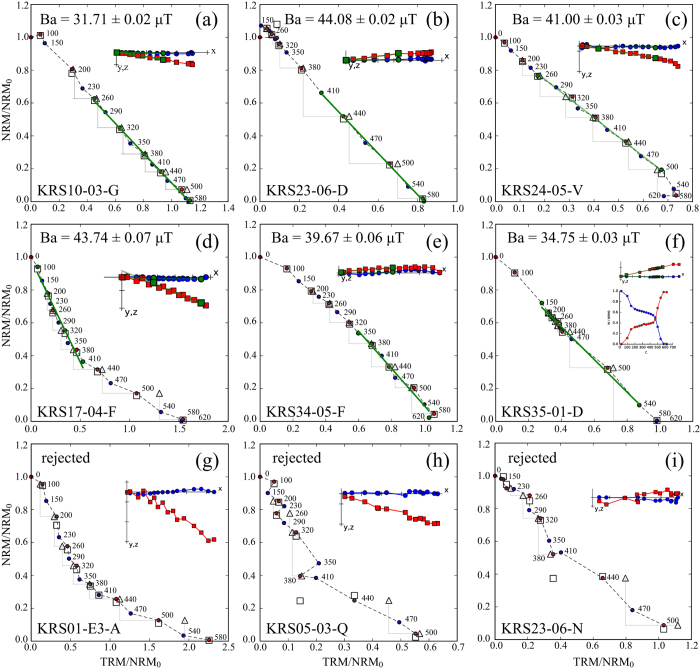
Examples of Arai diagrams. Examples of (**a**–**c**) ideal, (**d**–**f**) nearly ideal, and (**g**–**i**) non-ideal rejected Arai diagrams and together with their corresponding vector diagrams as insets. The green continuous line is the Principle Component Analysis (PCA), triangles are pTRM-checks, squares are tail-checks, red dots are ZI-steps, and blue dots are IZ-steps. In the vector diagram blue circles are the declination that is oriented parallel to the x-axis, and red squares are the inclination.

**Figure 2:**
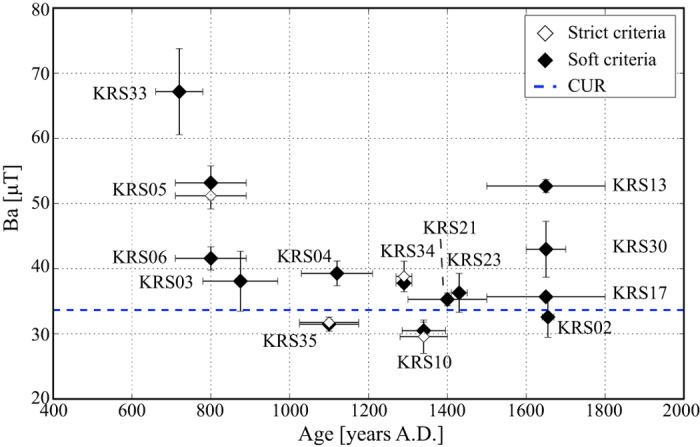
Comparison of the obtained results after application of the strict (white diamonds) and the soft selection criteria (black diamonds), and the current geomagnetic field value at Korsimoro (CUR).

**Figure 3:**
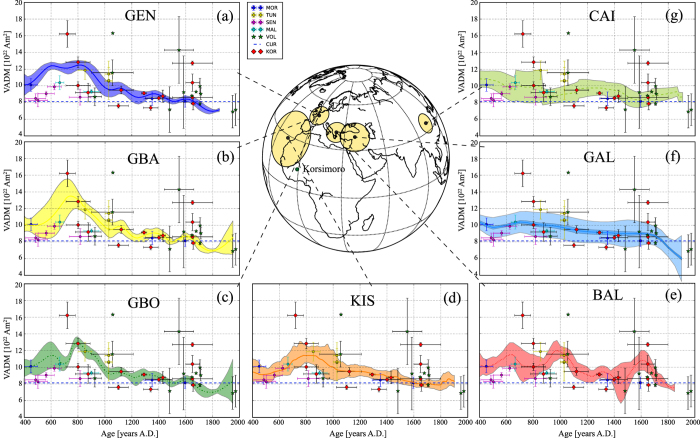
Virtual Axial Dipole Moments (VADMs) from Korsimoro compared to other data and palaeosecular variation curves. Data from Geomagia50.v3 database (MOR: Morocco, TUN: Tunisia, SEN: Senegal, MAL: Mali) from a radius of about 2500 km around Korsimoro. VOL: volcanic data from the same area. KOR: Data from this study, corrected for cooling rate. These data are compared to: (**a**) GEN: Western European curve^30^. (**b**) GBA: Bayesian curve for Western Europe^2^. (**c**) GBO: Bootstrap curve for Western Europe^2^. (**d**) KIS: SW-European/W-African curve^31^. (**e**) BAL: Balkan curve from Eastern Europe^1^,^30^. (**f**) GAL: Middle Eastern curve^32^. (**g**) CAI: Eastern Asian curve^33^. The circles on the map correspond to the areas from which data for the constructions of the PSV curves was selected. Also shown is CUR, the current VADM calculated for the latitude of Korsimoro. The map in this figure was produced with the Python 2.7.10 software (https://www.enthought.com).

Three types of Arai diagrams were observed: (1) an ideal linear behavior with one principle component in the vector diagrams (Fig. 1a–c); (2) nearly ideal behavior with two different slopes (Fig. 1d), slightly curved diagrams (convex and concave; Fig. 1e), and linear Arai and vector diagrams, but two phases visible in the demagnetization steps (NRM lost versus temperature) (Fig. 1f); (3) non-ideal behavior with a strong zigzag or chaotic behavior (Fig. 1g–i). Arai diagrams of type (3) were rejected, because they did not fulfill the selection criteria. In the case of specimens with two slopes in the Arai diagram we only chose the low temperature slope if the vector diagram shows a single linear component to the origin (Fig. 1d). However, in most cases the high-temperature part was selected. Start and end points of the PCA were, in general, chosen based on the quality criteria and on the agreement with sister specimens from the same furnace. Most Arai diagrams of specimens of T4 are slightly concave shaped, indicating multidomain grains dominating the magnetic mineralogy (Fig. 1g). Some specimens from furnaces KRS04, 21, 23, 23, and 35 have two phases in their demagnetization diagrams: the first unblocking temperature is at around 200 °C and the second at 580 °C. The unblocking temperature of 200 °C resembles the HCSLT phase, observed in samples from furnace KRS04 in 3IRM measurements^15^ (Fig. 1f). Demagnetization and remagnetization (pTRM versus temperature) are symmetrical in general, indicating a stable mineralogy. Vector diagrams of these specimens are linear with one component of magnetization while in many cases the corresponding Arai diagrams are as well linear.

### Results of Cooling rate and Anisotropy of ARM corrections

Results of the cooling rate (CR) experiment indicate that in eleven out of 42 cases |*r*_2_| > |*r*_1_|, which indicates that chemical alteration has occurred during the experiment. In these cases the correction values do not anymore reflect the original mineralogy. In total, four specimens have |*r*_1_| > 15%, of which three were rejected applying the soft criteria due to large scatter, which might indicate problems during the CR experiment. The forth specimen has its PCA fit to a temperature that is below its CR experiment temperature, because the Arai diagram shows alteration, but this is not reflected in the *r*_2_ parameter. After rejecting all values with |*r*_2_| > |*r*_1_| and |*r*_1_| > 15%, 26 values were used for CR correction. On average |*r*_1_| has values of (6.4 ± 1.0)%, |*r*_2_| = (2.1 ± 0.6)% and the CR correction factor *TRM*_1_/*TRM*_2_ = 0.88–1.17 with an average of 0.95 ± 0.01. For specimens without CR measurements, or if the CR correction was not accepted, we applied the CR correction average of the same samples. If this was not available, we applied the average of the furnace, or of the technique. The rational for this approach is that each furnace exhibits the same CR in all parts of the furnace, and furnaces from the same technique have similar characteristics, such as diameter and wall thickness. The CR corrections are similar to values measured by other authors, e.g., Morales *et al*.^19^ measured changes in intensity by 15% on brick samples, Chauvin *et al*.^20^ obtained typical values of 3–5% with maximum values of 10%, and Mitra *et al*.^11^ found very similar CR correction values between 0.85–1.09 with a median of 0.95. A CR correction factor greater than unity could indicate the presence of interacting SD or MD grains^21^. Another explanation for correction factors larger than unity might be that the cooling time of eight hours overestimates the natural cooling time. However, of all accepted CR correction factors only one is larger than unity.

Anisotropy of Anhysteretic Remanent Magnetization (AARM) measurements yielded changes of archaeointensities between (0.3–12.5)% and correction factors of (0.91–1.13). Five out of the 25 specimens, of which the AARM was measured, have larger changes in archaeointensity ranging from (6.0–12.5)%. Two are from KRS05 and three from KRS10. However, their sister specimens have low values. For the remaining 20 specimens factors are on average (1.6 ± 1.5)%, which indicates a negligible effect of anisotropy. For this reason, and because the correction factor depends on the orientation of the NRM within the field, and therefore is individual for each specimen, we do not correct for anisotropy. We investigated in more detail the five specimens with changes of archaeointensities >5%, to see if the correction of anisotropy improves the standard deviation. Specimens with larger anisotropy are from furnaces KRS05 (T1) and KRS10 (T3). For KRS05 the standard deviation increased after anisotropy correction, and therefore we prefer the values without anisotropy correction. For specimens from KRS10 the standard deviation gets smaller, but the furnace average does not change significantly. For reasons of consistency we do not correct these specimens neither for anisotropy. The obtained values of anisotropy correction agree with values found by other authors or are lower; e.g., Tema *et al*.^22^ obtained changes of <8% of archaeointensity due to ATRM correction in brick furnaces; Yamamoto *et al*.^23^ observed typical values between (5.9–12.0)% for baked clay from experimental furnaces; and Kovacheva *et al*.^24^ obtained corrections factor in general <5%, with maximum values of 11% on samples from baked clay from furnaces, burned soil, and bricks. Generally, the magnetic anisotropy of furnaces made of baked clay is not significant and its effect on the archaeomagnetic records may be considered negligible^24^,^25^,^26^, on the contrary to brick kilns or ceramics, where the magnetic anisotropy effect may be very important and its correction is necessary^20^,^27^,^28^. The five anisotropy values that are larger than the bulk of measurements may indicate, besides possible experimental problems, inhomogeneities in the furnace walls, because sister specimens have low values. These inhomogeneities might arise from recycled material, e.g., tuyeres, that have been used for the construction of some furnaces of T3. Furnaces built by other techniques are entirely made of clay, which contains small particles (mm to cm) of previously fired clay or of slag.

Final corrected furnace averages were calculated using a hierarchical approach, in which first sample and then furnace averages were calculated^29^ (Tables 3 and 4). After application of the soft acceptance criteria and the CR correction the average of KRS24 was rejected, because both *σ*_*Ba*_ (*μ*T and %) were not within the threshold values. Averages of KRS03 ad KRS33 passed only one of these values and were therefore accepted. The reason for using both *σ*_*Ba*_ as criterion is that archaeointensity values that are very low might not have *σ*_*Ba*_(%) accepted, or values that are very high might not have *σ*_*Ba*_(*μ*T) passing, and would therefore be rejected, although they are excellent determinations^18^. The average of KRS07 was rejected because only one specimen was accepted from this furnace. After application of the strict criteria and the CR correction three averages were rejected due to large standard deviations. The accepted and most reliable four furnace averages are from KRS05, 10, 34 and 35.

Averages per technique, corresponding to distinctive time periods, yielded the following outcome: T1: (50.0 ± 13.2) *μ*T, T2: (36.2 ± 4.2) *μ*T, T3: (34.0 ± 3.1) *μ*T, and T4: (41.0 ± 8.9) *μ*T. Technique averages of T2 and T3 are very similar and are well confined, while averages of T1 and T4 are higher and have larger standard deviations. Nevertheless, we approve these averages, because each technique spans at least 100 years, and may incorporate large geomagnetic field variation.

## Discussion

In general, the Korsimoro furnaces are very suitable for archaeointensity determinations due to the following observations: the main magnetic mineral carrying the magnetic signal is magnetite, with a small contribution of hematite and a high coercivity stable low temperature phase; furthermore, the majority of the specimens exhibit a single linear component during demagnetization with a small viscous component. The difference between the results of the soft and the strict selection criteria is very small (Fig. 2). The largest deviation of 2 *μ*T is observed for KRS05, while KRS35 stays about the same with a difference of 0.3 *μ*T. This outcome strengthens our results using the soft criteria and gives confidence to the selection of the limits for the PCA and the choice of reliable selection criteria. The weakest result is the one of KRS33, which has the largest standard deviation (Table 3). This analysis confirms that a selection of very strict criteria might lead to the rejection of reliable results^18^. Hence, we consider the results after applying the soft criteria as our principal results. After the application of the CR correction 13 out of 15 furnace averages have smaller standard deviations than the uncorrected values. Furnaces with the most confined archaeointensity results are KRS17, KRS21, and KRS34 (Table 3). The suitability of the samples is reflected in a relatively high acceptance rate on specimen level of about 64%.

The new archaeointensity data from Korsimoro are compared to other data from West Africa included in the Geomagia50.v3 database^7^. These data are very limited and come from Morocco, Tunisia, Senegal and Mali^8^,^9^,^10^,^11^ (Fig. 3). Comparison with several palaeosecular variation curves has also been made (Fig. 3a): the master curve for Western Europe^30^ (GEN; Fig. 3a), the Western European Bayesian and Bootstrap curves^2^ (GBA and GBO, respectively; Fig. 3b,c), the SW-European/W-African curve^31^ (KIS; Fig. 3d), the Balkan curve for Eastern Europe^1^,^30^ (BAL; Fig. 3e), the bootstrap intensity variation curve for the Middle East^32^ (GAL; Fig. 3f), and the Eastern Asian reference curve^33^ (CAI; Fig. 3g). Furthermore, we compare our data with volcanic data from the Canary Islands^14^,^31^,^34^ (Fig. 3).

Mitra *et al*.^11^ investigated pottery from Senegal and Mali and observed that their intensity data were lower than data from Egypt and Morocco. We observe as well similar values as data from Senegal and Mali (Fig. 3), but also agreement with volcanic data from Canary islands, which are located at higher latitudes. Additionally, we observe higher intensity values in Burkina Faso that are similar to or higher than data from Tunisia and Morocco. Furthermore, Mitra *et al*.^11^ found an intensity high prior to 700 AD. However, these data are with a maximum value of 43.4 *μ*T much lower than our largest value of 67.2 *μ*T. After 700 AD our data agree well with the sloping trend of GEN, KIS, GBO, GBA and GAL until 1300 AD.

Data from KRS33 at 720 AD exhibit a high intensity compared to other values from this period and coincide with the maximum in GBA (Fig. 3b). The higher value at 800 AD (KRS05) supports as well a rather elevated intensity similar to the maximum in the GBO, GEN, and KIS curves, and coincides with GBA. These rather high intensities at 720–800 AD agree very well with one of the most significant features of the variation of the geomagnetic field intensity during the past two millennia: Gomez-Paccard *et al*.^2^ reported a strong intensity maximum of up to about 90 *μ*T at 800 AD in Western Europe. This high intensity feature was first pinpointed in the work of Genevey *et al*.^27^ for data from France. Archaeomagnetic jerks, which were defined as concurrence of sharp cusps in direction and maxima in intensity^12^, were clearly observed at 200 AD and 1400 AD, and less well constrained at 800 BC and 800 AD, in data from Europe and the Middle East. The latter, less well constrained archaeomagnetic jerk is supported by our data. The differences between GBO and GBA at around 800 AD arise from a lack of data^2^. In the other W-European curves this feature seems to be rather shifted to younger or older ages and is in general weaker. However, this intensity peak is not observed in the Eastern European record of the BAL curve. The Middle Eastern curve is very smooth compared to the other curves, but has a slightly higher intensity at around 800 AD, while the East Asian curve has a sort of plateau between 650–850 AD. Our lower data at 800 AD (KRS06) agrees with data from Senegal and Mali^11^ and supports a lower field intensity similar to the Balkan and the Middle Eastern curve at this time.

Genevey *et al*.^30^ observed in their Western European master curve five prominent peaks in the last 1500 years, with a recurrence of 250 years. Some of our data coincide with the peaks in the master curve, e.g., at 800 AD and 1650 AD. However, data from Africa is still too scarce to observe a 250 years period.

At about 1650 AD we obtain four data points that range from 32.6–52.7 *μ*T (Table 3), with KRS02 and KRS17 coinciding with most of the reference curves, except for the Balkan curve that better agrees with the higher value of KRS30. However, the age ranges of these samples are very different, i.e., *σ*_*age*_ ranges from 15 to 150 years. The largest intensity at 1650 AD from KRS13 coincides as well with a maximum in the BAL curve and is in the error range of another high intensity value from the volcanic data. In most of the palaeosecular variation curves we observe a small local maximum with values up to about 37 *μ*T, while in the BAL curve there is a maximum at around 1600 AD with values up to 50 *μ*T. This might be a hint that it is a local geomagnetic field feature. Following the recent study of Donadini *et al*.^15^ directional features of Africa coincide with those from Europe. Our data agree as well with field features observed in both, East and West European reference curves, and with the East Asian curve at around 800 AD.

**Figure 4:**
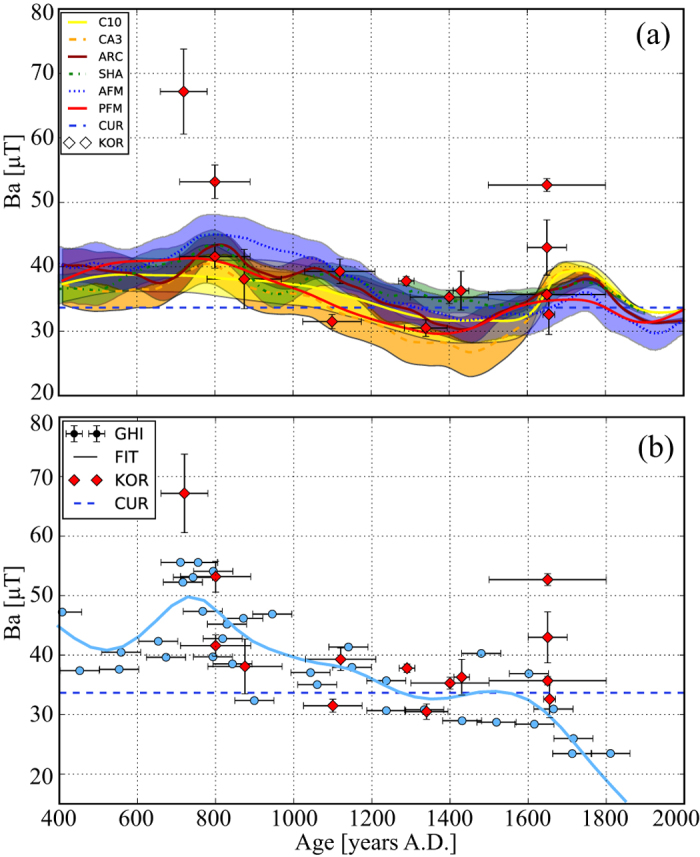
(**a**) Comparison of the Korsimoro intensity data (KOR) with geomagnetic field models. C10 is the CALS10k.1b models, CA3 the CALS3k.4 model, ARC the ARCH3k.1 model, SHA the SHA.DIF14k, AFM the Archeo FM model, PFM the pfm9k.1a model. For references please refer to the text. (**b**) Comparison to marine sediment data^38^ (GHI). The spline fit of the GHI data is also shown as continuous line. CUR is the current field strength.

Furthermore, in Fig. 4a, we compare our data to geomagnetic field models, such as the CALS10k.1b^6^, the CALS3k.4^35^, the ARCH3k.1^36^, the SHA.DIF14k^4^, the Archeo FM^5^, and the pfm9k.1a^37^. All models are calculated for the coordinates of Korsimoro. The models are much smoother and have less amplitude than the individual data and the palaeosecular variation curves. At around 800 AD the maximum present in all models coincides with our highest intensities. The sloping trend between 800–1500 AD is reflected in the declining KRS intensities. The broad maximum at 1700 AD appears to coincide with our data.

Finally, in Fig. 4b we compare KRS data to a marine sediment record from Cape Ghir (GHI), West Africa^38^. To facilitate the comparison we calculated a cubic smoothing spline fit with a smoothing factor of 5 (Fig. 4b). Since the marine sediment data are relative intensities we scaled the discrete data and the spline fit to make them comparable to our data. Therefore we divided the data and spline fit with their median and multiplied them with the median of the archaeomagnetic data from this study. Data from Bleil and Dillon^38^ agree very well with the KRS data in the whole time period, for example with the high intensities at 700–800 AD, as well with the lower intensity at 800 AD. Between 1400–1800 AD a local maximum is visible in the smoothing spline fit and discrete marine sediment data coinciding with increasing values at Korsimoro between 1300–1450 AD. From 1500 AD onward the marine sediment data decreases, while three KRS results are higher for the same period. However, the lowest intensities in this period (KRS02, KRS17, KRS30) agree with data from GHI.

## Conclusions

Our 17 new archaeointensity data, dated from 700 to 1700 AD offer a significant contribution to the reconstruction of the past geomagnetic field in Africa. The new data show an intensity peak around 700–800 AD, which seems to be a very interesting feature of the geomagnetic field in West Africa. More data from this period are necessary to confirm the occurrence of such high intensity values, already previously identified in Europe^2^. The selection of two different classes of selection criteria allows us to reinforce our archaeointensity results and offer a measure of determining more reliable results. Unfortunately, the available data from Africa are still too sparse to draw conclusions about similarities and differences between West African, European, and Asian field features. It has to be noted that this lack of data particularly influences the construction of reference curves and the reliability of the global models in this area. For this reason, the acquisition of new data from Africa and the southern hemisphere remains an important goal to better understand the existence of local geomagnetic field features generated at the core mantle boundary and to improve global geomagnetic field models.

## Methods

### Archaeological context and age determination

**Figure 5:**
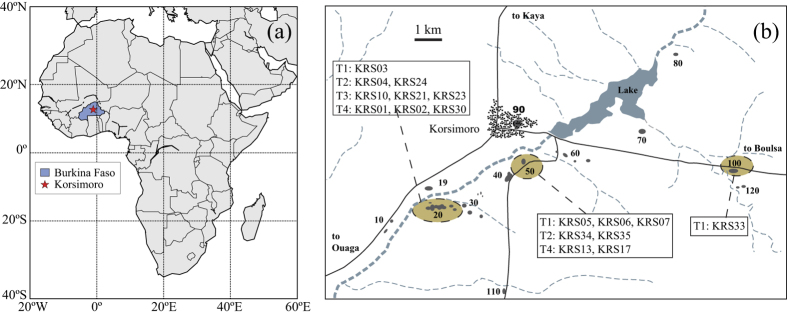
(**a**) Map of Africa with the location of Burkina Faso and the sampling site Korsimoro. The map was produced using the Python 2.7.10 software (https://www.enthought.com). (**b**) Schematic aerial view of the investigated sectors. Furnaces (KRS) that were selected for archaeointensity experiments are located in the encircled sectors. The different techniques (T1–T4) to which the furnaces belong are also indicated. Reprinted from Earth and Planetary Science letters, 430, Donadini, F., Serneels, V., Kapper, L., El Kateb, A., Directional changes of the geomagnetic field in West Africa: Insights from the metallurgical site of Korsimoro, 349–355, Copyright (2015), with permission from Elsevier.

Korsimoro is located at 12.79°N latitude and 1.09°W longitude in Burkina Faso, about 70 km North of the capital, Ouagadougou (Fig. 5a). Archaeological research has revealed several sectors of metallurgical activity, which are distributed over an area of 10 × 6 km with each sector spanning about 1 km (Fig. 5b). Four different types of smelting furnaces, corresponding to four production phases, have been identified^39^, hereafter referred to as techniques T1–T4. The types of furnaces were distinguished based on the smelting techniques, amount of slag production, grouping, typology of furnaces, type of slag deposits, tuyeres, and their chronology. Tuyeres are tubes, which served for the oxygen exchange during the smelting process. In order to confine the ages of the different production phases, radiocarbon (^14^C) age determinations were obtained from charcoal and burnt straw (Table 5). Samples for dating were collected from the bottom of the excavated furnaces, where possible, and at the basis of the slag deposits. Age determinations were analyzed in Beta Analytics laboratory and were calibrated using the IntCal09 database^40^,^41^,^42^,^43^ with a calibration method of Talma and Vogel^44^. For more details on the dating of the structures please refer to Serneels *et al*.^39^, Serneels *et al*.^45^ and Donadini *et al*.^15^. The combination of the archaeological age with the ^14^C datings defined four refined, subsequent time intervals corresponding to the four techniques, which cover a total time period of 700–1700 AD (Table 5).

**Figure 6:**
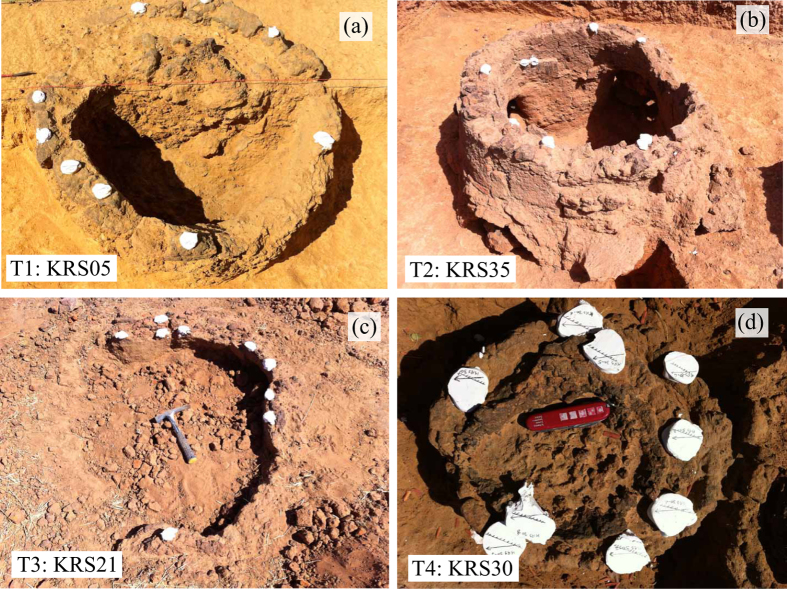
Examples of furnaces from each technique. (**a**) T1, (**b**) T2, (**c**) T3, and (**d**) T4. The plaster of Paris was used for the *in-situ* orientation of the samples.

Technique 1 (T1) comprises series of about ten aligned pits that are filled with slag. Each pit is the base of a furnace, which was used one time. It was covered with a movable shaft, which was displaced from pit to pit after each smelt. T1 is considered as the phase of minor activity with an annual production of <100 kg of iron (Fig. 6a). Technique 2 (T2) was used during the main iron production phase. Archaeologists found large areas of up to 500 m^2^, which are covered by a slag layer of about 50 cm thickness. The annual production of iron was about 30 000 kg and was produced in furnaces of about 1 m in diameter, which were used several times (Fig. 6b). Furnaces of technique 3 (T3) were found around areas of T2. During this phase a major technological change took place, in which the slag was tapped out of the furnace. Furnaces are surrounded by annular piles of slag. The annual iron production sums up to 10 000 kg (Fig. 6c). Smelting furnaces of technique 4 (T4) are small, with a diameter of about 30 cm. More than 800 of these furnaces were found clustered in small groups. This period is again characterized by minor smelting activity (Fig. 6d). An extended report on characteristics and illustrations of each technique are presented by Serneels *et al*.^39^ and Donadini *et al*.^15^.

### Sampling and methods

Overall 32 furnaces were sampled at Korsimoro for archaeomagnetic investigation, of which 17 were used for archaeointensity determinations (Fig. 5b). From each furnace 7–9 oriented block samples were collected. The orientation of the *in-situ* samples was recorded using plaster of Paris by marking the sun’s shadow on the plaster’s flat surface (Fig. 6). Each block sample was cut in cubes of 2 cm side length. In order to determine the rock magnetic properties Donadini *et al*.^15^ performed measurements of thermomagnetic curves (magnetic susceptibility versus temperature), hystereses, backfield curves, and 3-axes Isothermal Remanent Magnetization (3IRM) curves. Additionally, the samples were demagnetized thermally (TH) and with alternating fields (AF).

For the present study, archaeointensities were determined on 120 specimens, divided into three sets, using the IZZI-Thellier-Thellier protocol^46^. The first two sets of 26 and 52 specimens, respectively, were measured at the Laboratory for Natural Magnetism (LNM) of the ETH Zurich, Switzerland. The third set includes 42 specimens and was measured at the National Archaeomagnetic Service of the UNAM, Campus Morelia, Mexico. The 16 applied temperature steps range from 100 °C to 620 °C, or until specimens were completely demagnetized, with pTRM- and tail-checks after each second temperature step. Samples were heated in an ASC TD48 and TD48-SC thermal demagnetizer in the laboratories in Switzerland and Mexico, respectively. Magnetization was measured on a 2G Enterprises, model 755R, 3-axis DC-SQUID rock magnetometer in Switzerland and an Agico JR6 magnetometer in Mexico. Archaeomagnetic data was analyzed using the PmagPy-3.4.0 software^47^.

A cooling rate (CR) experiment was performed following the method presented by Chauvin *et al*.^20^ on the specimens of the third set. The experiment was performed at the temperature at which the specimens lost about 70% of their initial NRM, i.e. at 380 °C and 500 °C, for 14 and 27 specimens, respectively. In this experiment specimens were first cooled fast in the presence of a laboratory field of 35 *μ*T, in 30–45 minutes using the ventilator, acquiring a thermoremanent magnetization *TRM*_1_. Then the specimens were slowly cooled from the specific temperature in the same field and acquired *TRM*_2_. And finally, the first step was repeated to obtain *TRM*_3_. For the slow cooling step we applied two different approaches: (1) specimens that were heated to 380 °C were cooled ‘naturally’ by switching off the ventilator and by waiting that they reach room temperature in about eight hours; (2) for specimens heated to 500 °C we implemented a cooling rate protocol following Newton’s law of cooling, *dT*/*dt* = −*k(T* − *T*_0_), with temperatures *T* = 500 °C, *T*_0_ = 25 °C, the constant *k* = 0.5 and *t* the time, in which a natural cooling time of six hours was assumed based on archaeological considerations. In this protocol the temperature in the oven was adjusted every 15 minutes to follow the non-linear curve. The rational for the second approach is based on the empirical evidence that the cooling in the oven from 500 °C with switched-off ventilator presents a very fast exponential drop of the temperature (*t*_1/2_ ≈ 1.5 hrs), whereas in our approach the temperature decay is regulated as to follow a natural cooling time. This decay is still exponential, but with a cooling time closer to the ancient cooling behavior. Coefficients *r*_1_ = (*TRM*_2_ − *TRM*_1_)/*TRM*_1_ and *r*_2_ = (*TRM*_1_ − *TRM*_3_)/*TRM*_1_, which were presented by Chauvin *et al*.^20^, were calculated. Coefficient *r*_1_ measures the effect of the CR, while *r*_2_ the changes of the TRM acquisition capacity. Cooling rate corrections were rejected if |*r*_2_| > |*r*_1_| and |*r*_1_| > 15%. Archaeointensity values were corrected with the ratio *TRM*_1_/*TRM*_2_.

The magnetic anisotropy of the studied samples and its possible effect on the archaeointensity results has been investigated through the measurement of the anisotropy of anhysteretic remanent magnetization (AARM). Mitra *et al*.^11^ showed that the AARM tensor can be considered interchangeable to the anisotropy of TRM (ATRM) tensor in order to investigate and correct for anisotropy effects on archaeological materials. Selkin *et al*.^48^ have also proposed that the AARM measured after the Thellier experiment is a suitable measure of the remanence anisotropy. AARM and ATRM display similar properties for SD magnetites and are believed to be carried by the same grain fraction^49^. Determining the AARM has the advantage that additional alteration during heating at high temperatures is avoided^11^. We measured the AARM of 25 specimens from the first set, at the ALP Palaeomagnetic laboratory (Peveragno, Italy). For the calculation of the AARM tensor a 12 positions scheme has been followed, in order to eliminate any residual field^28^. The ARM was given using a constant bias field of 35 *μ*T produced by a small coil inside and a 30 mT peak field with the AF demagnetizing coil. After each position, the ARM was measured with a JR6 spinner magnetometer (Agico). The anisotropy tensor was calculated using the AREF program based on Tema^28^, and the correction factor with the method of Veitch *et al*.^50^.

## Additional Information

**How to cite this article:** Kapper, L. *et al*. Reconstructing the Geomagnetic Field in West Africa: First Absolute Intensity Results from Burkina Faso. *Sci. Rep.* **7**, 45225; doi: 10.1038/srep45225 (2017).

**Publisher's note:** Springer Nature remains neutral with regard to jurisdictional claims in published maps and institutional affiliations.

## Figures and Tables

**Table 1:** Threshold values of the soft and strict acceptance criteria on specimen level.

	*β*	DRATS	FRAC	MAD (°)	DANG (°)	*N*_*PCA*_	SCAT
Soft	≤0.085	≤15	≥0.5	≤7	≤8	≥6	—
Strict	≤0.08	—	≥0.7	≤5	≤5	≥10	True

*β* is the ratio of the standard error of the slope to the absolute value of the slope; DRATS the difference ratio sum; FRAC the fraction of remanence; MAD the maximum angular deviation; DANG the deviation of the angle, as to ensure that the characteristic component was chosen; *N*_*PCA*_ the number of points used for the principal component analysis (PCA); and SCAT the scatter parameter.

**Table 2:** Threshold values of the soft and strict acceptance criteria on furnace level.

	*σ*_*Ba*_ (*μ*T)	*σ*_*Ba*_ (%)	*N*_*Spec*_
Soft	≤5	≤10	≥2
Strict	≤3	≤8	≥3

*σ*_*Ba*_ is the standard deviation calculated in *μ*T and %; and *N*_*Spec*_ is the minimum number of specimens used to calculate the averages.

**Table 3:** Archaeointensity results averaged for each furnace after the application of the soft selection criteria.

Furnace	Age (yrs AD)	Tech.	*N*_*acc*_/*N*_*tot*_	*Ba* ± *σ*_*Ba*_ (*μ*T)	*σ*_*Ba*_ (%)	*Ba*_*cr*_ ± *σ*_*Ba*_ (*μ*T)	*σ*_*Ba*_ (%)
KRS33	720	T1	3/3	70.7 ± 7.0	9.9	67.2 ± 6.6	9.9
KRS05	800	T1	8/16	56.1 ± 2.9	5.2	53.2 ± 2.6	4.8
KRS06	800	T1	3/3	43.8 ± 1.9	4.3	41.6 ± 1.8	4.3
KRS07*	800	T1	1/3	—	—	—	—
KRS03	875	T1?	2/6	40.0 ± 4.8	12.0	38.1 ± 4.6	12.0
KRS35	1100	T2	12/13	34.5 ± 0.7	1.9	31.5 ± 1.1	3.6
KRS24*	1120	T2	4/9	33.1 ± 7.2	21.9	30.7 ± 7.8	25.4
KRS04	1120	T2	4/4	42.3 ± 2.0	4.8	39.3 ± 1.9	4.8
KRS34	1290	T2	9/12	40.3 ± 0.7	1.8	37.8 ± 0.7	1.8
KRS10	1340	T3	8/8	32.2 ± 1.4	4.4	30.5 ± 1.3	4.4
KRS21	1400	T3	4/6	36.4 ± 1.0	2.8	35.3 ± 1.0	2.8
KRS23	1430	T3	9/12	38.8 ± 3.1	7.9	36.3 ± 3.0	8.3
KRS13	1650	T4	3/3	55.4 ± 1.1	1.9	52.7 ± 1.0	1.9
KRS30	1650	T4	2/3	45.3 ± 4.5	10.0	43.0 ± 4.3	10.0
KRS17	1650	T4	2/3	37.6 ± 0.8	2.1	35.7 ± 0.7	2.1
KRS02	1655	T4	3/7	34.3 ± 3.2	9.4	32.6 ± 3.1	9.4

‘Tech.’ indicates the technique, *N*_*acc*_/*N*_*tot*_ is the number of accepted versus the total number of specimens, *Ba* is the furnace average, *σ*_*Ba*_ the standard deviation, *Ba*_*cr*_ is the archaeointensity after cooling rate correction. Furnaces with stars were rejected because their standard deviation *σ*_*Ba*_ > 5 *μ*T and >10%. All other furnaces that are not in this table were rejected because they did not pass the quality criteria. The age of KRS03 was radiocarbon dated to T1, but it could not be related archaeologically with certainty to T1.

**Table 4:** Archaeointensity results averaged for each furnace after the application of the strict selection criteria.

Furnace	Age (yrs AD)	Tech.	*N_acc_*/*N_tot_*	*Ba* ± **σ*_*Ba*_* (*μ*T)	**σ*_*Ba*_* (%)	*Ba*_*cr*_ ± **σ*_*Ba*_* (*μ*T)	**σ*_*Ba*_* (%)
KRS33*	720	T1	3/3	68.7 ± 9.9	14.5	65.2 ± 9.4	14.5
KRS05	800	T1	6/16	53.9 ± 2.1	3.9	51.2 ± 2.0	3.9
KRS06*	800	T1	3/3	48.2 ± 11.3	23.5	45.8 ± 10.7	23.5
KRS35	1100	T2	6/13	34.2 ± 0.2	0.7	31.8 ± 0.5	1.6
KRS34	1290	T2	6/12	41.3 ± 2.5	6.0	38.8 ± 2.4	6.1
KRS10	1340	T3	4/8	31.2 ± 2.7	8.6	29.6 ± 2.6	8.6
KRS23*	1430	T3	7/12	42.3 ± 6.1	14.4	39.5 ± 5.2	13.2

‘Tech.’ indicates the technique, *N*_*acc*_/*N*_*tot*_ is the number of accepted versus the total number of specimens, *Ba* is the furnace average, *σ*_*Ba*_ the standard deviation, *Ba*_*cr*_ is the archaeointensity after cooling rate correction. Furnaces with stars were rejected because their standard deviation *σ*_*Ba*_ > 3 *μ*T and >8%. All other furnaces not included in this table were rejected because they did not pass the quality criteria.

**Table 5:** Investigated furnaces, their calibrated ages, and the age error given as 2-*σ* bounds ‘*σ*_*age*_’.

Nr.	Furnace	Age (yrs AD)	*σ*_*age*_ (yrs)	Dat.	Tech.	*N_samp_*/*N_spec_*	Sec.
1	KRS33	720	60	^14^C	T1	1/3	100
2	KRS05	800	90	^14^C	T1	5/16	50
3	KRS06	800	90	arch.	T1	2/3	50
4	KRS07	800	150	arch.	T1	1/3	50
5	KRS03	875	95	^14^C	T1	4/6	20
6	KRS35	1100	75	^14^C	T2	4/13	50
7	KRS04	1120	90	^14^C	T2	3/4	20
8	KRS24	1120	90	arch.	T2	3/9	20
9	KRS34	1290	20	^14^C	T2	3/12	50
10	KRS10	1340	55	^14^C	T3	4/8	20
11	KRS21	1400	100	arch.	T3	2/6	20
12	KRS23	1430	20	^14^C	T3	4/12	20
13	KRS13	1650	150	arch.	T4	1/3	50
14	KRS17	1650	150	arch.	T4	1/3	50
15	KRS30	1650	50	arch.	T4	1/3	20
16	KRS01	1640	20	^14^C	T4	5/9	20
17	KRS02	1655	15	^14^C	T4	3/7	20

‘Dat.’ indicates the dating method used: ‘^14^C’ denotes kilns that were dated by radiocarbon and ‘arch.’ denotes an age determination based on radiocarbon dates from the same context and archaeological constraints; ‘Tech.’ indicates the technique; ‘*N*_*samp*_’ the number of samples investigated, ‘*N*_*spec*_’ the total number of specimens per kiln; and ‘Sec.’ the sector.
